# Altered innate immune profile in blood of systemic mastocytosis patients

**DOI:** 10.1002/clt2.12167

**Published:** 2022-06-14

**Authors:** Alba Pérez‐Pons, María Jara‐Acevedo, Ana Henriques, Paula Navarro‐Navarro, Andrés C. García‐Montero, Iván Álvarez‐Twose, Carlos E. Pedreira, Laura Sánchez‐Muñoz, Daniela Damasceno, Carolina Caldas, Javier I. Muñoz‐González, Almudena Matito, Juan Flores‐Montero, Oscar González‐López, Ignacio Criado, Andrea Mayado, Alberto Orfao

**Affiliations:** ^1^ Cancer Research Center (IBMCC, USAL‐CSIC) Department of Medicine and Cytometry Service (NUCLEUS) Universidad de Salamanca Salamanca Spain; ^2^ Centro de Investigación Biomédica en Red Cáncer (CIBERONC) Madrid Spain; ^3^ Biomedical Research Institute of Salamanca Salamanca Spain; ^4^ Spanish Network on Mastocytosis (REMA) Toledo and Salamanca Spain; ^5^ Sequencing Service (NUCLEUS) Universidad de Salamanca Salamanca Spain; ^6^ Instituto de Estudios de Mastocitosis de Castilla La Mancha (CLMast) Virgen del Valle Hospital Toledo Spain; ^7^ Systems and Computing Department (PESC) COPPE Federal University of Rio de Janeiro (UFRJ) Rio de Janeiro Brazil

**Keywords:** cytokines, dendritic cells, microenvironment, monocyte, systemic mastocytosis

## Abstract

**Background:**

Mast cells (MC) from systemic mastocytosis (SM) patients release MC mediators that lead to an altered microenvironment with potential consequences on innate immune cells, such as monocytes and dendritic cells (DC). Here we investigated the distribution and functional behaviour of different populations of blood monocytes and DC among distinct diagnostic subtypes of SM.

**Methods:**

Overall, we studied 115 SM patients ‐ 45 bone marrow mastocytosis (BMM), 61 indolent SM (ISM), 9 aggressive SM (ASM)‐ and 32 healthy donors (HD). Spontaneous and *in vitro*‐stimulated cytokine production by blood monocytes, and their plasma levels, together with the distribution of different subsets of blood monocytes and DCs, were investigated.

**Results:**

SM patients showed increased plasma levels and spontaneous production by blood monocytes of IL1β, IL6, IL8, TNFα and IL10, associated with an exhausted ability of LPS + IFNγ‐stimulated blood monocytes to produce IL1β and TGFβ. SM (particularly ISM) patients also showed decreased counts of total monocytes, at the expense of intermediate monocytes and non‐classical monocytes. Interestingly, while ISM and ASM patients had decreased numbers of plasmacytoid DC and myeloid DC (and their major subsets) in blood, an expansion of AXL^+^ DC was specifically encountered in BMM cases.

**Conclusion:**

These results demonstrate an altered distribution of blood monocytes and DC subsets in SM associated with constitutive activation of functionally impaired blood monocytes and increased plasma levels of a wide variety of inflammatory cytokines, reflecting broad activation of the innate immune response in mastocytosis.

## 1 INTRODUCTION

Systemic mastocytosis (SM) comprises a heterogeneous group of disorders that range from bone marrow (BM) mastocytosis (BMM) and indolent SM (ISM) to advanced forms of SM (AdvSM), such as aggressive SM (ASM), SM with an associated hematological neoplasm (SM‐AHN) and mast cell leukaemia (MCL).^1^ ^,^ ^2^ Most (>90%) SM cases carry the *KIT*D816 V mutation in the tyrosine kinase domain of the stem cell growth factor receptor gene (*KIT*), while other *KIT* mutations are present in a smaller percentage (≈1%–4%) of cases.^3^ *KIT*D816 V (and other *KIT* mutations) induce constitutive activation of *KIT* and its downstream Ras/MAP/ERK, PI3 K/AKT, JNK and STAT3 signaling pathways,^4^ ^,^ ^5^ leading to an expansion of activated clonal mast cells (MC) and an increased release of MC mediators (e.g. tryptase and histamine), and inflammatory cytokines by the activated MC.^6^, ^7^, ^8^, ^9^, ^10^

MC are myeloid cells that play key roles in the innate/inflammatory immune response.^11^ Once activated, MC interact with other immune cells either by direct cell‐to‐cell contact or via release of mediators that act on surrounding immune cells, including monocytes, macrophages, dendritic cells (DC), T‐cells and endothelial cells.^12^ MC‐released serine proteases such as α‐tryptase, cleave receptors ‐e.g. PAR2 (protease‐activated receptor‐2) and EMR2 (epidermal growth factor‐like module–containing mucin‐like hormone receptor–like 2)‐ expressed on the surface membrane of human monocytes and macrophages, leading to their downstream activation.^13^, ^14^ In parallel, other MC mediators such as tumour necrosis factor‐alpha (TNFα) and prostaglandin D2 (PGD2) also activate macrophages and instruct DC to polarize TCD4^+^ cells toward a T‐helper 2 (Th2) response, respectively.^15^, ^16^ Those effects of MC mediators on surrounding immune cells are further enhanced by activated MC‐derived exosomes which have been shown to induce in vitro phenotypic and functional maturation of DC,^17^ and directly promote activation, proliferation and cytokine secretion ‐e.g., interleukin (IL)‐22, interferon (IFN)‐γ and TNFα‐by TCD4^+^ cells following HLA class‐II and CD40‐CD154‐mediated signaling.^18^ Based on this immunomodulatory role of MC, it might be hypothesized that constitutive activation of *KIT* in SM MC, may lead to an altered distribution and/or functional behaviour of other immune cells, with further impact on dysregulated immune responses and disease behavior.^19^, ^20^, ^21^

In this regard, increased evidences support an altered innate and adaptative immune response in SM. Thus, abnormally decreased TCD8^+^ and NK‐cell counts together with increased levels of circulating type II innate lymphoid cells (ILC2) have been reported in ISM,^22^, ^23^ in parallel to an increase in plasma of IL6 and other inflammatory cytokines, which would further contribute to an enhanced activation and accumulation of clonal MC in SM.^24^ Despite these findings, the major (cell) source of these cytokines and their pathogenic role in the outcome of SM, remain poorly understood. Thus, Tobio et al.^25^ identified MC from two SM patients as the main source of IL6,^25^ while Teodosio et al. reported low IL6 gene expression levels in highly‐purified BM MC from both ISM and ASM patients, who typically had increased IL6 plasma levels.^26^ ^,^ ^27^ Overall, the latter findings suggest that immune cells other than MC, capable of producing IL6 and other inflammatory cytokines, might be key in enhancing immune dysregulation in SM.^27^ Among candidate cells, blood monocytes, tissue macrophages and DC are included.

Here we investigated the distribution and functional behavior of different populations of blood monocytes and DC in a large series of 115 SM patients. Our ultimate goal was to gain insight into the potential alterations of innate myeloid cells in blood of patients with distinct diagnostic subtypes of SM.

## 2 MATERIAL AND METHODS

### 2.1 Patients, controls and samples

A total of 115 SM patients diagnosed according to the WHO criteria^2^ at the reference centres of the Spanish Network on Mastocytosis (REMA; Mast Cell Unit, Hospital del Valle, Toledo; and, Cancer Research Centre, Salamanca, Spain), were studied. Those included 45 BMM, 61 ISM and 9 ASM cases. Thirty‐two age‐matched healthy adult donors (HD) who had no past or present history of allergy or other immune disorders, were studied as controls. Demographic, biochemical and clinical data on SM patients grouped according to the diagnostic subtype of the disease are shown in Table 1 in Supplementary Information and their distribution per each set of assays performed is detailed in Table 2 in Supplementary Information. Briefly, functional analysis of spontaneous (ex vivo) cytokine production was initially investigated at the single‐cell level in 53 patients −20 BMM, 28 ISM and 5 ASM‐ and 16 HD; further in vitro monocyte‐stimulatory assays were performed in a subset of 7 of these 53 patients −2 BMM, 4 ISM and 1 ASM‐ and 15 HD. Quantification of cytokine plasma levels was performed in parallel in 52 patients −19 BMM, 28 ISM, and 5 ASM‐ and 6 HD‐ and flow cytometry immune cell profiling studies were done in 70 patients −24 BMM, 39 ISM and 7 ASM‐ and 12 HD; finally, soluble plasma levels of CCL2 (C‐C motif chemokine ligand‐2) and EMR2 plus CXCR2 (C‐X‐C motif chemokine receptor‐2), SAA1 (serum amyloid‐A1) and TLR2 (Toll Like Receptor‐2) were evaluated in 57 ‐20 BMM, 32 ISM and 5 ASM‐ and in 30 patients −9 BMM, 17 ISM and 4 ASM‐, respectively.

Each patient and HD gave his/her written informed consent to participate in the study according to the Declaration of Helsinki. The study was approved by the local Ethics Committees (approval codes: PI 2019 03 260 and CEIC 2016 09 07).

### 2.2 Spontaneous and *in vitro*‐stimulated cytokine production by blood monocytes

For ex vivo cytokine production assays, blood was diluted 1:1 (vol:vol) in RPMI 1640 and incubated with 10 mg/ml brefeldin A (BFA) (Sigma‐Aldrich, St Louis, MO) for 6 h at 37°C in a 95% humid atmosphere containing 5% CO_2_. Cultured cells were washed in phosphate buffered saline (PBS) and incubated for 30 min in the darkness (4°C) with a 12‐color combination of monoclonal antibodies (MAb) directed against CD45, CD33, CD123, CD3, CD19, CD56, CD16, FcɛR1α, HLA‐DR, CD300e and CD14, for identification and subsetting of PB monocytes (Table 3 in Supplementary Information). Stained cells were washed in PBS, fixed for 15 min with solution A of Fix & Perm (eBioscience, San Francisco, CA), washed in PBS, permeabilized with solution B of Fix & Perm, and stained for 15 min (RT) in the dark in separate aliquots for intracytoplasmic IL1β, IL6, IL8, IL10, IL12, IL13, TNFα, and TGFβ (Table 3 in Supplementary Information). Stained cells were washed in PBS and measured (>2 × 10^5^ leukocytes) in a BD‐LSR Fortessa X‐20 flow cytometer (BD), using the FACSDiva software (BD). For data analysis, the Infinicyt software (Cytognos SL, Salamanca, Spain) was used to evaluate cytokine production by each subset of blood monocytes identified, based on the percentage of positive cells/cytokine.

In a subset of patients and of HD, an aliquot of blood diluted 1:1 (vol:vol) in RPMI 1640 was cultured in parallel for 2 h in the presence of (20 μg/mL) lipopolysaccharide (LPS) (Sigma‐Aldrich) and (0.4 μg/mL) IFNγ (R&D Systems, Minneapolis, MN) to actively induce cytokine production in vitro, before adding BFA to block cytokine secretion to the extracellular compartment. Stimulated cells were then cultured for another 4 h, and stained as described above. In all experiments, an aliquot of unstimulated blood was processed in parallel as negative control.

### 2.3 Quantification of cytokine levels in plasma

Soluble TNFα, IL1β, IL6, IL8, IL10 and IL12 levels were assessed in plasma using the Cytometric Bead Array (CBA) immunoassay system (BD) and the Human Inflammatory Cytokine Kit (BD), strictly following the recommendations of the manufacturer. Afterward, a minimum of 3000 events per bead population was measured in a FACSCanto II flow cytometer (BD) using FACSDiva™. For data analysis the FCAP Array software (v3.0; BD) was used.

### 2.4 Flow cytometric identification and enumeration of blood monocyte and dendritic cell populations

The distribution of distinct subsets of monocytes and DC was investigated in K3‐EDTA‐anticoagulated fresh (<24 h) blood. For this purpose, 10^7^cells/sample were stained with the Euroflow 14‐color (16‐antibody) monocyte‐DC immune monitoring tube (MoDC IMM tube) (Supplementary Table 3) using the EuroFlow Bulk Lysis standard operating procedure for staining of cell surface membrane markers‐only (www.euroflow.org), as previously described.^28^, ^29^, ^30^ In a subset of SM patients and HD, expression of EMR2 (CD312) and the IL8‐receptor (CD182) on different subsets of stained blood monocytes and DC were also evaluated (Table 3 in Supplementary Information), after measuring ≥5 × 10^6^ nucleated cells/sample in a 3‐laser Aurora flow cytometer (Cytek, Landing Parkway, CA) equipped with the SpectroFlo software (Cytek). For data analysis Infinicyt was used.

### 2.5 Quantification of CCL2, CXCR2, EMR2, SAA1 and TLR2 plasma levels

CCL2 and EMR2 plasma levels were measured using the Human CCL2/MCP‐1 Quantikine (R&D Systems), and the Human EMR2 (Thermo‐Fisher, Waltham, MA) enzyme‐linked immunoabsorbent assay (ELISA) kits. CXCR2, SAA1 and TLR2 plasma levels were evaluated with the Human CXCR2 (EIAab Science Inc, Wuhan, Hubei, China), SAA1 (Abcam, Cambridge, UK), and TLR2 (Cloud Clone Corp., Wuhan, Hubei, China) ELISA kits, respectively.

### 2.6 Statistical methods

For all continuous variables, median (range) and mean (standard deviation; SD) values, as well as the 10th, 25th, 75th and 90th percentiles, were calculated; for categorical variables, frequencies were used. The statistical significance of differences observed among groups was assessed with the Kruskal‐Wallis and the Mann‐Whitney *U* tests (for continuous variables), or the chi‐square and Fisher exact tests (for categorical variables). The degree of correlation between different variables was determined by the Spearman's test. For all statistical analyses the IBM‐SPSS software (version 23.0; IBM, Armonk, NY) was used. *p*‐values < 0.05 were considered to be associated with statistical significance.

## 3 RESULTS

### 3.1 Spontaneous ex vivo cytokine production by blood monocytes and cytokine plasma levels in SM

**FIGURE 1:**
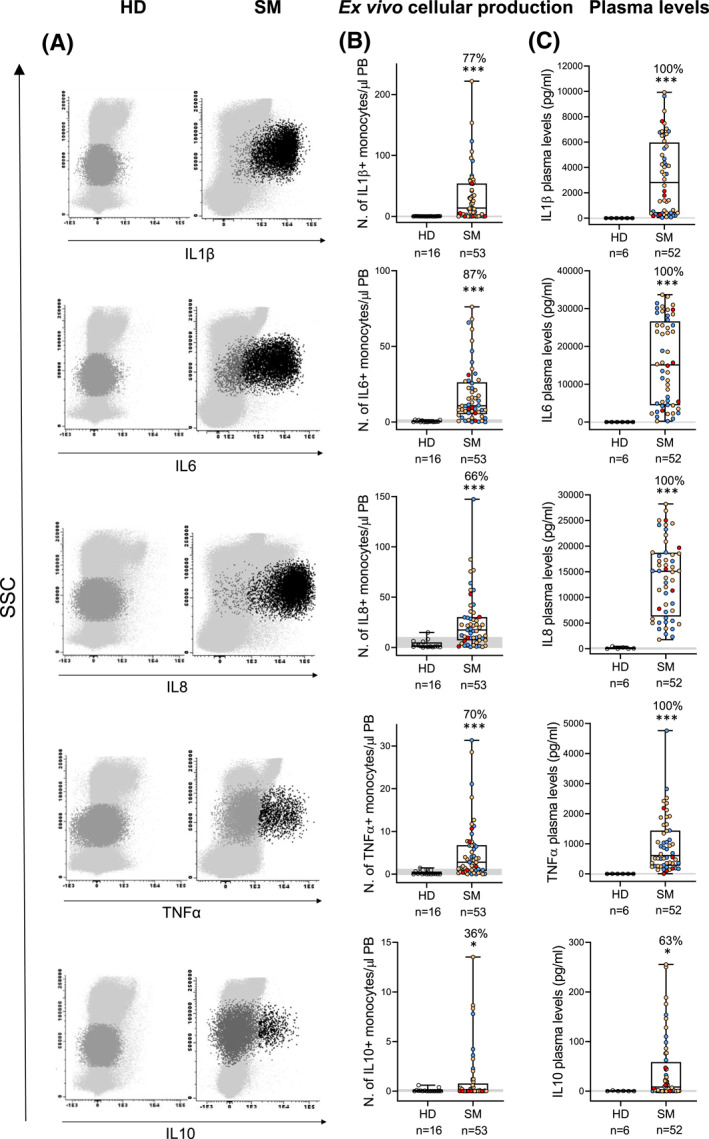
Distribution of ex vivo cytokine‐producing blood monocytes and soluble cytokine levels in plasma of systemic mastocytosis (SM) patients versus age‐matched healthy donors (HD). (A) Bivariate flow cytometry dot plot histograms showing representative intracytoplasmic staining profiles for IL1β, IL6, IL8, IL10 and TNFα by cytokine producing blood monocytes (black dots) from a representative SM patient (right panels) and an HD (left panels). (B) Number of ex vivo cytokine‐producing monocytes in blood of SM patients versus HD. (C) Soluble cytokine plasma levels in SM versus HD. Notched‐boxes extend from 25th to 75th percentile values, the lines in the middle and vertical lines correspond to median values and the 10th and 90th percentiles, respectively, while dots represent each individual case analyzed. Blue dots represent bone marrow mastocytosis cases, orange dots show indolent systemic mastocytosis patients, and red dots corresponded to aggressive systemic mastocytosis cases. Percentage values indicate the percentage of SM patients above percentile 95 of HD. *p*‐values: * <0.05, ** <0.010, *** <0.001. ASM, aggressive systemic mastocytosis; BMM, bone marrow mastocytosis; ISM, indolent systemic mastocytosis; SSC, sideward light scatter

**FIGURE 2:**
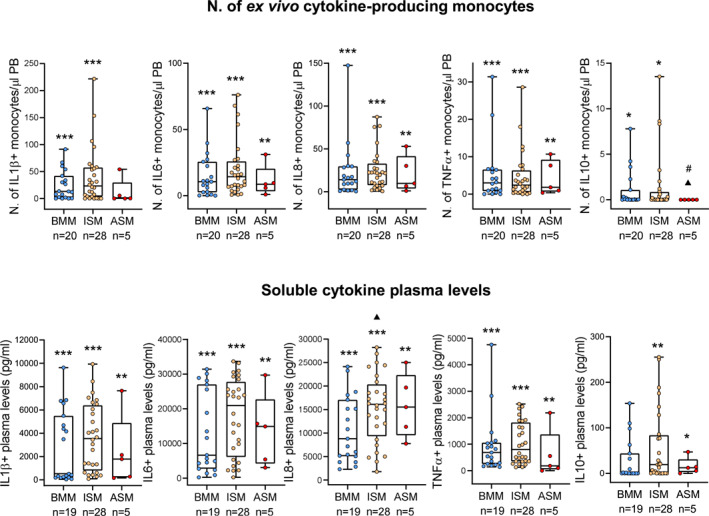
Distribution of ex vivo cytokine‐producing monocytes in blood (A) and soluble cytokine levels in plasma (B) of SM patients classified according to the distinct diagnostic subtypes of the disease. Notched‐boxes extend from 25th to 75th percentile values, and the lines in the middleand vertical lines correspond to median values and the 10th and 90th percentiles, respectively, while dots represent each individual case analyzed. * versus HD, ▲ versus BMM, # versus ISM. *p*‐values: * <0.05, ** <0.010, *** < 0.001, ▲ <0.05, ▲▲ <0.01, ▲▲▲ < 0.001, # <0.05; ## <0.01, ### < 0.001. ASM, aggressive systemic mastocytosis; BMM, bone marrow mastocytosis; ISM, indolent systemic mastocytosis

Under baseline ex vivo conditions, blood monocytes from SM patients showed a significantly (*P* < 0.001) increased (vs. HD) spontaneous production of IL1β, IL6, IL8, TNFα, and to a less extent also IL10 (*P* = 0.036), with higher numbers of cytokine‐producing monocytes (Figure 1A,B). Of note, increased numbers of IL6^+^, IL8^+^ and TNFα^+^ monocytes in blood after short‐term in vitro culture (*P* ≤ 0.017) were observed across all diagnostic subtypes of SM (Figure 2A). In turn, increased numbers of IL1β^+^ and IL10^+^ monocytes were restricted to BMM (*P* ≤ 0.018) and ISM (*P* ≤ 0.034) while normal in ASM (Figure 2A). In contrast, ISM cases displayed decreased counts in blood of TGFβ^+^ monocytes (*P* = 0.03 vs. HD) (Figure 1B in Supplementary Information), while similar percentages of IL12^+^ and IL13^+^ monocytes were found in blood of SM and HD (Figure 1A in Supplementary Information).

To determine whether the increased spontaneous production of inflammatory cytokines by blood monocytes was associated with increased amounts in plasma of the same cytokines, due to their in vivo release to the extracellular medium, we subsequently, investigated their plasma levels. Interestingly, increased IL1β, IL6, IL8, TNFα (*P* < 0.001) and IL10 (*P* = 0.024) levels were found in plasma of SM versus HD (Figure 1C), both groups showing similarly low/undetectable IL12 levels (Figure 1B in Supplementary Information). Of note, the same pattern of alteration was observed for soluble IL1β, IL6, IL8 and TNFα (*P* ≤ 0.006) across the distinct diagnostic subtypes of SM, although ISM patients displayed higher IL8 levels than BMM cases (*P* = 0.05), and increased IL10 levels were restricted to ISM and ASM patients (*P* = 0.010 and *P* = 0.015 vs. HD, respectively) (Figure 2B). These data suggest that spontaneously increased cytokine production by circulating blood monocytes occurs in SM, which might lead to increased levels in plasma of the same inflammatory cytokines. In fact, a significant correlation between the number of IL1β^+^ (*P* = 0.009), IL6^+^ (*P* = 0.043) and IL8^+^ (*P* = 0.016) blood monocytes and the plasma levels of the corresponding cytokines was observed (Figure 1C in Supplementary Information).

**FIGURE 3:**
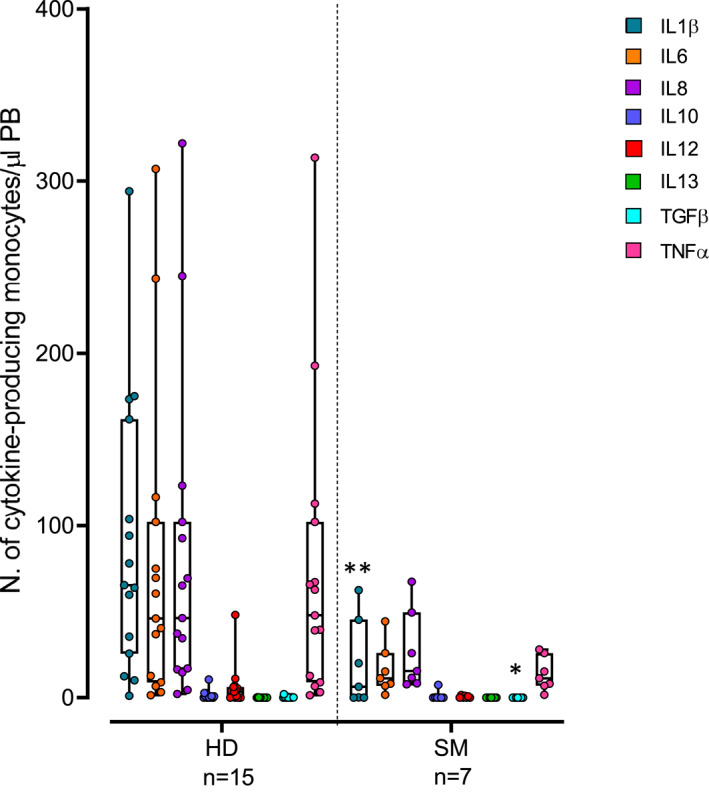
Number of cytokine‐producing monocytes after in vitro stimulation of blood samples with LPS plus IFNγ in systemic mastocytosis (SM) patients classified according to the diagnostic subtypes of the disease versus age‐matched healthy donors (HD). Notched‐boxes extend from 25th to 75th percentile values, the lines in the middle and vertical lines correspond to median values and the 10th and 90th percentiles, respectively, while dots represent each individual case analyzed. *p*‐values: * <0.05, ** <0.010, *** <0.001

In order to further assess whether increased spontaneous production of inflammatory cytokines, was associated with an overall enhanced cytokine production capacity after in vitro stimulation, blood monocytes from 7 SM patients and 15 HD were further stimulated in vitro with LPS plus IFNγ, prior to evaluation of cytokine production. Interestingly, in vitro stimulated blood monocytes from SM patients showed lower numbers of for example, IL1β^+^ (*P* = 0.008) and TGFβ^+^ monocytes (*P* = 0.013) than HD, although for the latter there were hardly any producing cells (Figure 3). These findings suggest that (compared to normal monocytes), circulating monocytes from SM patients are constitutively activated, but functionally exhausted. Alternatively, they might consist of different subsets which are for example, unresponsive to LPS and IFNγ (Figure 3 in Supplementary Information).

### 3.2 Distribution of distinct populations of monocytes and dendritic cells in blood of SM patients

**FIGURE 4:**
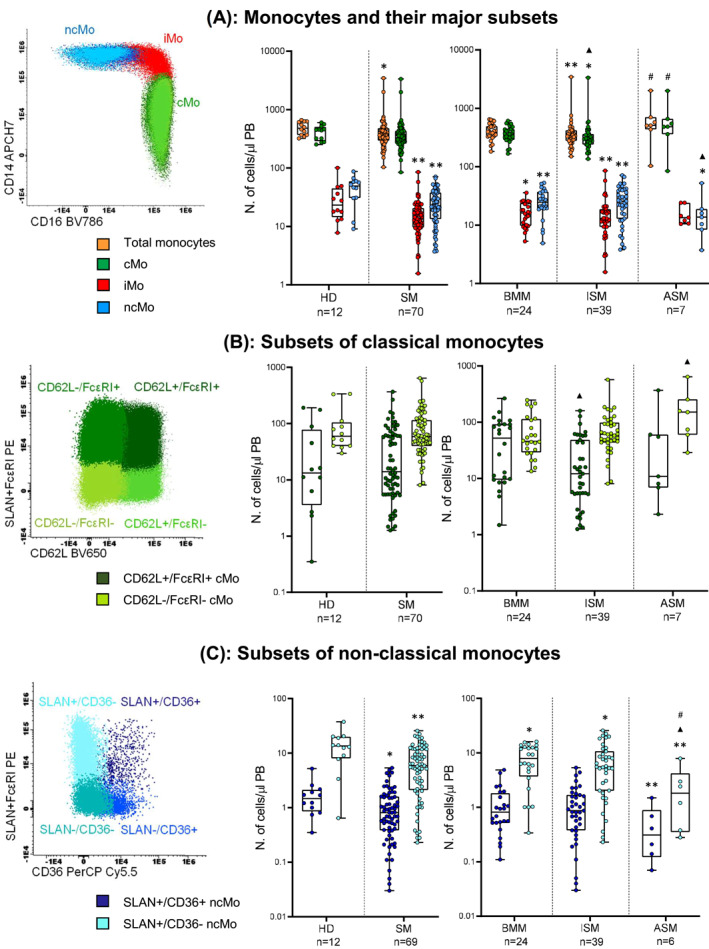
Distribution of total monocytes and the different populations of classical (cMo), intermediate (iMo) and non‐classical (ncMo) monocytes in blood (left panel) of systemic mastocytosis (SM) patients and healthy donors (HD). A, Distribution of total monocytes and their major subsets of iMo, cMo and ncMo in HD versus all SM patients (middle panel) grouped per disease subtype (right panel). B, Distribution of different substets of cMo (CD62L^+^/FcɛRI^+^ cMo and CD62L^−^/FcɛRI^−^ cMo) in blood of HD versus SM (middle panel) distributed according to the diagnostic subtype of the disease (right panel). C, Distribution of different subsets of ncMo (SLAN^+^/CD36^+^ and SLAN^+^/CD36^−^ ncMo) in HD versus SM patients (middle panel) classified according to the distinct diagnostic subtypes of the disease (right panel). Notched‐boxes extend from 25th to 75th percentile values, the lines in the middle and vertical lines correspond to median values and the 10th and 90th percentiles, respectively, while dots represent each individual case analyzed. * versus HD, ▲ versus BMM, # versus ISM. *p*‐values: * <0.05, ** <0.010, *** < 0.001, ▲ <0.05, ▲▲ <0.01, ▲▲▲ < 0.001, # <0.05, ## <0.01, ### < 0.001. BMM, bone marrow mastocytosis; ISM, indolent systemic mastocytosis; ASM, aggressive systemic mastocytosis

Compared to HD, total monocyte counts were significantly decreased (*P* = 0.032) in SM. This was at the expense of both intermediate monocytes (iMo) (*P* = 0.008) and non‐classical monocytes (ncMo) (*P* = 0.002) and, within the latter, of the SLAN^+^CD36^+^ (*P* = 0.037) and SLAN^+^CD36^−^ (*P* = 0.008) ncMo subsets. ISM patients also showed decreased counts of classical monocytes (cMo) versus both HD (*P* ≤ 0.021) and other SM patients (*P* = 0.043 vs. BMM; and *P* = 0.019 vs. ASM) (Figure 4A). Of note, decreased cMo counts in ISM versus BMM were mostly at the expense of the CD62 L^+^ FcεRI^+^ subset (*P* = 0.011), while ASM cases showed higher counts of CD62L^−^ FcεRI^−^ cMo versus BMM (*P* = 0.047) (Figure 4B).

Among SM, BMM and ISM were those groups of patients that showed significantly decreased counts of iMo versus HD (*P* = 0.041) (Figure 4A), while ncMo and their SLAN^+^CD36^−^ subset were decreased (vs. HD) across all subtypes of SM (Figures 4A,C). In turn, ncMo were more pronouncedly decreased in ASM than BMM (*P* = 0.047), in association with lower counts of their SLAN^+^CD36^−^ subset –vs BMM and ISM (*P* ≤ 0.047) (Figure 4C).

**FIGURE 5:**
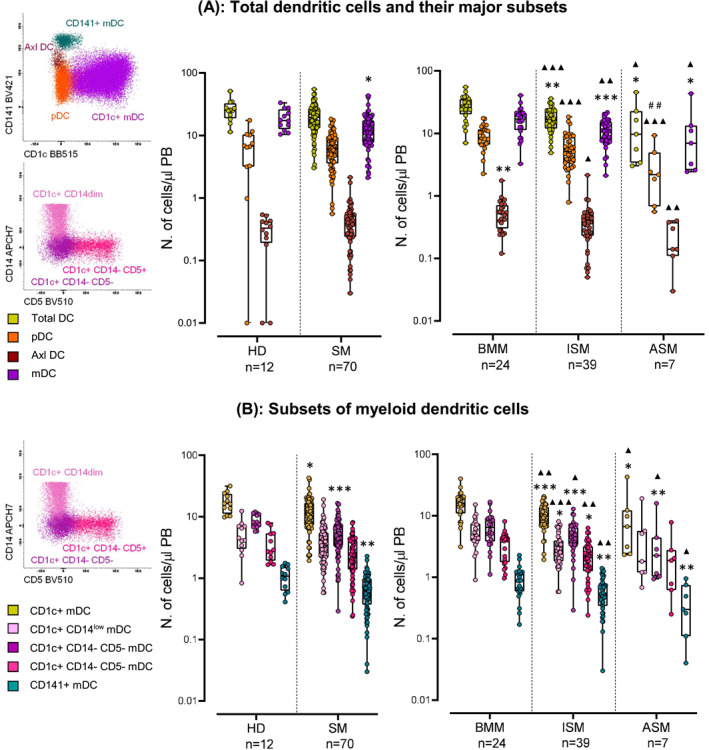
Distribution of different populations of dendritic cells (DC) (A) and myeloid DC subsets (B) in blood (left column in A‐B) from healthy donors (HD) versus systemic mastocytosis (SM) patients (middle panels in A‐B) classified according to the distinct diagnostic subtypes of the disease (right panels in A‐B). Notched‐boxes extend from 25th to 75th percentile values, the lines in the middle and vertical lines correspond to median values and the 10th and 90th percentiles, respectively, while dots represent each individual case analyzed. * versus HD, ▲ versus BMM, # versus ISM. *p*‐values: * <0.05, ** <0.010, *** < 0.001, ▲ <0.05, ▲▲ <0.01, ▲▲▲ < 0.001, # <0.05, ## <0.01, ### < 0.001. ASM, aggressive systemic mastocytosis; BMM, bone marrow mastocytosis; ISM, indolent systemic mastocytosis

Likewise, blood counts of myeloid DC (mDC) and their CD1c^+^, CD1c^+^CD14^−^CD5^−^ and CD141^+^ mDC subsets, were all decreased in SM (*P* ≤ 0.015 vs. HD) (Figure 5A,B). Among SM patients, decreased total DC and mDC (and their subset) counts were found in ISM and ASM patients versus both HD (*P* ≤ 0.035) and BMM (*P* ≤ 0.038) (Figure 5A,B). Such decrease was due to lower counts of the CD1c^+^ CD14^low^ and CD1c^+^ CD14^−^CD5^+^ mDC subsets in ISM versus HD (*P* ≤ 0.030) and BMM (*P* ≤ 0.005) (Figure 5B). In turn, the number of plasmacytoid DCs (pDCs) progressively decreased from BMM to ISM (*P* = 0.001 vs. BMM) and ASM cases (*P* = 0.019 vs. ISM) (Figure 5A). Conversely, Axl DC were increased only in BMM cases (*P* ≤ 0.01 vs. HD; ISM; and ASM) (Figure 5A). Altogether, these results reveal markedly altered monocyte and DC kinetics in blood of SM, in association with their functional alteration (Figure 3 SuppInfo).

### 3.3 Plasma levels and surface membrane expression of senescence‐, chemotaxis‐ and activation‐associated markers on circulating monocytes in SM

**FIGURE 6:**
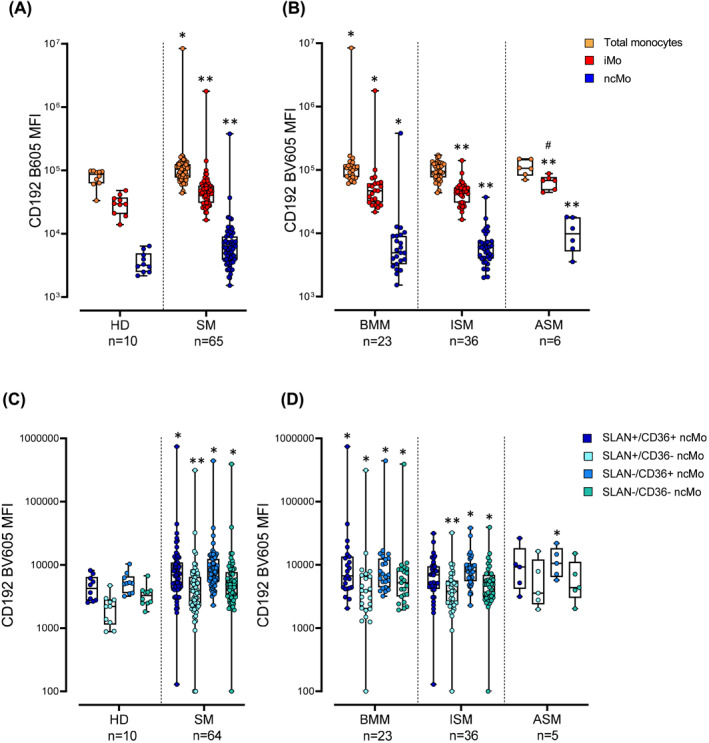
Surface membrane expression levels of the CD192/CXCR2 chemokine receptor on circulating blood monocytes and their subsets in healthy donors (HD) versus systemic mastocytosis (SM) patients (A and C) distributed according to the diagnostic subtype of the disease (B and D). Notched‐boxes extend from 25th to 75th percentile values, the lines in the middle and vertical lines correspond to median values and the 10th and 90th percentiles, respectively, and the dots represent each individual case studied. * versus HD, # versus ISM. *p*‐values: * <0.05, ** <0.010, *** < 0.001, # <0.05, ## <0.01, ### < 0.001. ASM, aggressive systemic mastocytosis; BMM, bone marrow mastocytosis; ISM, indolent systemic mastocytosis; MFI, mean fluorescence intensity (arbitrary units scaled from 0 to 4,194,304)

Normal CXCR2 and SAA1 levels were found in plasma of SM (Figure 2C,E in Supplementary Information), associated with undetectable TLR2 and normal CXCR2 membrane expression levels on blood monocytes from SM patients and HD. In contrast, lower levels of CCL2 were found in plasma of ISM versus both BMM and ASM (*P* ≤ 0.042) (Figure 2B in Supplementary Information), together with increased cell surface expression of its CD192 receptor on monocytes (*P* = 0.037) of SM patients, regardless of the subtype of the disease. This was at the expense of higher CD192 levels on iMo and ncMo (*P* ≤ 0.003) ‐including their SLAN^−^CD36^+^, SLAN^−^CD36^−^, SLAN^+^CD36^−^, and SLAN^+^CD36^+^ (*P* ≤ 0.034) subsets‐ (Figure 6A,B). Thus, CD192 expression was increased (*P* ≤ 0.046) in all subsets of ncMo in BMM, all ncMo subsets except SLAN^+^CD36^+^ ncMo of ISM patients, and only on SLAN^−^CD36^+^ ncMo in ASM cases (Figure 6B). Altogether these findings suggest that except for CCL2/CD192, all other markers investigated in SM, which are associated with immunosenescence, show normal soluble and cellular expression levels. Likewise, normal plasma and cell surface membrane expression levels of the EMR2 activation‐associated marker were observed on blood monocytes of SM patients, suggesting a limited role for the tryptase‐EMR2 pathway on the activation of blood monocytes.

## 4 DISCUSSION

Mast cells are key players in various physiologic and pathologic immune conditions due to their functional plasticity and the ability to release a broad array of bioactive mediators.^31^, ^32^, ^33^ Thereby, MC might exert their effects on other immune cells via their ability to modulate tissue distribution and functionality of lymphocytes, monocytes, macrophages, DC and endothelial cells, among other cells^12^ ^,^ ^34^ ^,^ ^35^ As an example, tryptase can activate normal blood mononuclear cells and induce them to synthesize and release pro‐inflammatory cytokines (e.g. TNFα, IL1β and IL6), but not Th1 (IL12 and INFγ) or Th2 cytokines (IL4 and IL10).^36^ In this regard, Li et al. confirmed that monocytes are the source of IL6 secreted in response to tryptase.^37^

In line with these observations, previous studies in SM have recurrently reported abnormally high cytokine levels in plasma.^25^, ^27^, ^38^, ^39^ Interestingly, these have been repeatedly suggested to derive from an increased production by the pathologic MC.^25^ Despite this, data on cytokine production by purified primary MC could not confirm an increased production of inflammatory cytokines at the mRNA level.^26^ In turn, most of the above studies have specifically focused on cytokine production by MC, while little is known about the potential contribution of other immune cells to the increased levels of inflammation cytokines in SM. Recently, Hermans et al. reported upregulation of proteins associated with the IL6, TLR4, TNFα and IFN type I/II signaling pathways in a small group of eight ISM patients. In parallel, they also found decreased percentages of pDC and monocytes together with an increased proportion of Th2 cells in another group of 18 ISM patients, suggesting signs of broad immune activation, beyond, MC in SM.^38^ However, functional analysis of the numerically altered immune cells is still missing.

Here, we investigated the potential existence of functional and numerical alterations on blood circulating monocytes and DC in patients with distinct diagnostic subtypes of SM. Overall, our results revealed that increased plasma levels of inflammatory cytokines, such as IL1β, IL6, IL8 and TNFα and to less extent IL10, but not IL12, IL13 and TGFβ, is a hallmark of SM, from BMM and ISM to ASM. Further single cell ex vivo studies demonstrated a parallel increase in the spontaneous production of the same cytokines by blood monocytes in SM, the number of cytokine‐producing IL1β^+^, IL6^+^ and TNFα^+^ cells significantly correlating with their corresponding plasma levels. Although a primary functional alteration of blood monocytes might exist in SM, we could not relate it with the presence of *KIT* D816V mutated monocytes (Supporting Information). Alternatively, spontaneous in vivo activation and functional impairment of circulating blood monocytes could be a consequence of an underlying MC activation and/or MC immunosenescence. In this regard, the presence of *KIT‐*mutated MC is a hallmark of SM, which has been associated with an increased release of tryptase and others mediators by *KIT‐*mutated MC. Tryptase is known to activate cells that express EMR2 and/or PAR2 on their surface membrane such as human monocytes, EMR2‐induced monocyte activation triggering inflammation.^13^ Despite this, here we failed to demonstrate altered plasma and/or monocyte cell surface expression levels of the EMR2 and PAR2 receptors (data not shown), suggesting no direct link between the tryptase‐EMR2/PAR2 pathway and activation of blood monocytes in SM. This is further supported by the lack of correlation found here between the altered (cellular and soluble) levels of inflammatory cytokines and both serum tryptase levels and the *TPSAB1* genotype, of BMM, ISM and ASM patients (Supporting Information). Similarly, all senescence associated markers investigated (CXCR2, SAA1 and TLR2) showed normal soluble and cellular expression levels, suggesting activated blood monocytes from SM patients are pro‐inflammatory rather than immunesenescent cells.

Despite blood monocytes from SM patients showed an abnormally increased spontaneous production of inflammatory cytokines, these cells appeared to be functionally exhausted, after in vitro stimulation with LPS and IFNγ. However, it should be noted that LPS plus IFNγ induce (conventional) activation of innate cells and polarize macrophages towards an M1‐like pro‐inflammatory phenotype^40^, ^41^ while Th2 cytokines such as IL4 and IL13 are also activators of monocytes^42^, ^43^, ^44^ able to polarize these cells towards an M2‐like anti‐inflammatory phenotype. Consequently our findings might also reveal a different response profile of blood circulating monocytes from SM patients to different stimuli. Further in vitro functional studies in which the response of blood monocytes from SM patients to other stimuli such as to Th2 cytokines are needed to better understand the biological and pathophysiological significance of our findings.

The altered functionality of blood monocytes here reported in SM could also be due to a redistribution of different populations of monocytes in blood with increased numbers of activated monocytes (and potentially also DC). Here we demonstrated that SM patients do exhibit important numerical alterations of blood circulating monocyte and DC populations, in addition to their functional activation. Thus, we confirmed previous data showing decreased total monocyte and DC counts in SM.^38^ Decreased monocyte counts were at the expense of both iMo and specific (SLAN^+^CD36^−^ and SLAN^+^CD36^+^) subsets of ncMo. Despite these alterations were consistent across distinct diagnostic subtypes of SM, important differences were also noted among BMM, ISM and ASM cases. Thus, ISM patients showed lower counts of total monocytes and cMo than ASM cases. In contrast, ncMo (and their SLAN^+^CD36^−^ subset) decreased in all subtypes of SM, particularly among ASM patients. Overall, these results point out the potential existence of an increased recruitment of blood monocytes ‐particularly of iMo and terminally differentiated (SLAN^+^) ncMo‐ to tissues such as the skin where activated clonal MC accumulate. In line with this hypothesis, previous data^45^ has shown that tumour HMC1 cells produce high levels of monocyte chemoattractant protein‐1 (MCP‐1 or CCL2), at the same time in vitro activated MC recruit monocytes and induce them to produce inflammatory cytokines such as TNFα.^45^ Fully in line with this, here we also demonstrated that blood monocytes from SM patients display increased levels on their surface membrane of the high‐affinity MCP‐1/CCL2 receptor, CCR2 (CD192) associated with lower CCL2 plasma levels.

Similarly to monocytes, pDC, mDC, and particularly the CD1c^+^, CD1c^+^CD14^−^CD5^−^ and CD141^+^ mDC subsets, were also numerically decreased in blood in SM and expressed increased amounts of CD192 on their surface membrane. These findings suggest that DC might also undergo increased migration to tissues where clonal MC accumulate. Altogether, these results support and extend on previous observations^38^ suggesting that clonal MC might produce proteases that affect the skin environment and activate keratinocytes to produce CCL19, in addition to MCP‐1/CCL2, (a chemokine that specifically recruits pDC to tissues) with potential consequences on activation of T‐cells.^38^ Interestingly, decreased counts of total DC, pDC and mDC (and their subsets) were more pronounced in ISM and ASM versus BMM, while Axl‐DC were specifically increased in the latter patient group. At present, little is known about Axl‐DC. Despite this, Axl‐DC have been recently shown to promote T cell proliferation, and to be increased in inflammed tissues such as the lung and skin^46^, ^47^, ^48^, ^49^ Altogether, these data suggest that Axl DC might play a specific role in patients presenting with severe MC activation symptoms (e.g. anaphylaxis), a hallmark of BMM. In line with this hypothesis, our data also suggest a close association between higher counts of AXL DC counts, as well as virtually all subsets of DC and monocytes circulating in blood of SM patients, and the occurrence of anaphylaxis (Table 4 in Supplementary Information).

In summary, here we confirm and extend on previous observations in SM, revealing an altered distribution of specific subsets of circulating monocytes and DC, associated with significantly increased numbers of activated but functionally exhausted blood monocytes, leading to increased levels of pro‐inflammatory cytokines in plasma. Taken together, these findings point out a broad immune activation in SM, with a potential role for innate myeloid cells other than MC in the pathophysiology and clinical behaviour of mastocytosis; the specific mechanisms involved deserve further investigations.

## AUTHOR CONTRIBUTION

**Alba Pérez‐Pons**: Formal analysis; Methodology; Writing – original draft. **Maria Jara‐Acevedo**: Formal analysis; Investigation; Supervision. **Ana Henriques**: Data curation; Methodology; Supervision. **Paula Navarro‐Navarro**: Formal analysis; Methodology; Supervision. **Andres Garcia‐Montero**: Funding acquisition; Investigation; Writing – review & editing. **Ivan Alvarez‐Twose**: Data curation; Methodology; Supervision. **Carlos Pedreira E**: Methodology; Software. **Laura Sanchez Munoz**: Data curation; Methodology; Supervision. **Daniela Damasceno**: Methodology; Supervision. **Carolina Caldas**: Methodology; Supervision. **Javier I Muñoz‐Gonzàlez**: Formal analysis; Methodology; Supervision. **Almudena Matito**: Data curation; Methodology; Supervision. **Juan Flores‐Montero**: Methodology; Supervision. **Oscar Gonzàlez‐López**: Methodology; Supervision. **Ignacio Criado**: Methodology. **Andrea Mayado**: Conceptualization; Investigation; Project administration; Supervision; Writing – original draft; Writing – review & editing. **Alberto Orfao**: Conceptualization; Investigation; Project administration; Supervision; Writing – original draft; Writing – review & editing.

## CONFLICTS OF INTEREST

Authors declared no conflicts of interest.

## Supporting information

Supplementary Information 1

## Data Availability

The data that support the findings of this study are available from the corresponding author upon reasonable request.
