# Supplementary material for: Altered innate immune profile in blood of systemic mastocytosis patients
Source: Clin Transl Allergy. 2022 Jun 14;12(6):e12167. doi: 10.1002/clt2.12167 (PMC9194602; doi:10.1002/clt2.12167)
Supplement: Supplementary file 1 — Supplementary Information 1 [file CLT2-12-e12167-s001.docx]

**Supporting Information:**

**Material and Methods SuppInfo:**

**Serum tryptase levels.** Serum baseline tryptase (sBT) level was measured using the Phadia ImmunoCAP Tryptase System (Phadia/Thermo Fisher Scientific Inc., Uppsala, Sweden), according to the instructions of the manufacturer.

***KIT* mutational analysis**. The *KIT* D816V mutation was investigated in whole BM DNA and FACS-purified (≥97% purity) BM MC, eosinophils, monocytes, neutrophils, CD34^+^ hematopoietic precursors cells (HPC) and T cells from all 115 SM patients studied, as well as on purified PB monocytes (FACSAria, BD) from a subgroup of 17 SM patients. For *KIT* mutational analyses both an allele-specific oligonucleotide quantitative PCR (ASOqPCR) approach and a peptide nucleic acid (PNA)-mediated PCR-clamping method, were used (50)^,^ (51) in whole BM and purified cell DNA. The *KIT* D816V variant allele frequency (VAF) was determined in BM as previously described (52)(28)^,^ (53).

***TPSAB1* gene analysis**. Genotyping was performed in 103 SM patients by quantitative real-time digital PCR (qdPCR) following previously described methods (54); for this purpose the Biomark™ HD system (Fluidigm, South San Francisco, CA) and the Digital PCR Analysis Software (Fluidigm) were used. Hereditary α tryptasemia (HαT) was defined when 3 or more copies of the α tryptase gene copies or 2 copies of the α-tryptase gene together with in presence of 3 copies of the β-tryptase gene were found, following previously established criteria.

**Results SuppInfo.**

**A Spontanous *ex vivo* cytokine producing blood monocytes B Cytokine levels in plasma**

**Association between the presence of symptoms and both the distribution of monocyte and dendritic cell subsets in blood of SM patients.** In a subset of 70 SM patients, we further investigated if the presence of symptoms such as anaphylaxis, osteoporosis, pruritus or gastrointestinal symptoms would have an effect on the distribution of PB monocytes and DC. Thus, SM patients with anaphylaxis showed higher monocytes (P=.029) and DC counts (P<.001) vs SM cases without anaphylaxis, at the expense of all subsets of monocytes (P≤.040) and DC (P=<.002) (Table 4 SuppInfo). In addition, SM patients who had pruritus displayed decreased counts (P=.034) (vs cases without prutitus) of ncMo. In contrast, no statistically significant differences were found neither in the distribution of cytokine producing blood monocytes, nor in plasma levels of the cytokines investigated between SM patients presenting with or without anaphylaxis, pruritus, GI symptoms and/or osteoporosis (Table 4 SuppInfo).

**Association between the presence of the** ***KIT* D816V mutation in PB monocytes of SM patients and both the distribution of monocyte and dendritic cell subsets in blood and their cytokine production profile.** In a subset of 25 SM patients, including 10 BMM and 15 ISM, we further investigated the presence of the *KIT* D816V mutation in highly-purified blood monocytes by ASO-qPCR. *KIT* D816V^+^ blood monocytes were detected in 3/10 (30%) BMM and in 11/15 (73%) ISM patients (Table 5 SuppInfo). Of note, no statistically significant differences were found in the distribution of blood monocyte and DC populations, neither in their cellular and plasma cytokine levels, between cases that showed *KIT* D816V mutated vs unmutated blood monocytes (Table 5 SuppInfo). Thus, the presence of *KIT* D816V mutation in blood monocytes from SM patients might not have a direct effect on the altered distribution and the dysregulated cytokine production of blood monocytes and dendritic cells here observed among SM patients.

**Association between the presence of HαT and both the distribution of monocyte and dendritic cell subsets in blood and their cytokine production profile.** In a subset of 103 SM patients, including 40 BMM, 54 ISM and 9 ASM, we further investigated the *TPSAB1* genotype (Table 6 SuppInfo). Overall, the HαT genotype was detected in 7/40 (18%) BMM, 3/54 (6%) ISM and in 3/9 (33%) ASM patients (Table 6 SuppInfo). Of note, HαT patients showed increased numbers of total monocytes, cMo and mDC compared to patients showing a normal *TPSAB1* genotype (P=.036, P=.02 and P=.045, respectively).

| **Variable**  **Table 1 SuppInfo. Adult systemic mastocytosis patients (n=115): demographic, clinical and laboratory findings** **at diagnosis according to distinct subtypes of the disease.** | **SM**  **(n=115)** | **BMM**  **(n=45)** | **ISM**  **(n=61)** | **ASM**  **(n=9)** | **P**  **value** |
| --- | --- | --- | --- | --- | --- |

Results expressed as number of cases (percentage) or as^*^mean ± one standard deviation. SM, systemic mastocytosis; BMM, bone marrow mastocytosis; ISM, indolent systemic mastocytosis; ASM, aggressive systemic mastocytosis; y, years; M, male; F, female; LDH, lactate dehydrogenase; SAP, serum alkaline phosphatase; BM, bone marrow; MC, mast cell; FCM, flow cytometry; NS, statistically not significantly different (P>0.05); ND, not determined; † BMM vs ISM patients, ‡ BMM vs ASM patients, § ISM vs ASM patients, ¶ BMM, ISM and ASM patients.

| **Age (y)*** | 55±11 | 53±9 | 55±11 | 60±12 | NS |
| --- | --- | --- | --- | --- | --- |
| **Sex (M/F)** | 58/57  (50.4%/49.6%) | 29/16  (64%/36%) | 25/36  (41%/59%) | 4/5  (44%/56%) | 0.017^†^ |
| **Anaphylaxis** | 59 (52%) | 40 (89%) | 18 (30%) | 1 (11%) | <0.001^†‡^ |
| **Organomegaly** | 18 (16%) | 3 (6.7%) | 8 (13%) | 7(78%) | <0.001^‡§^ |
| **Haemoglobin (x10^9^l)*** | 14±1.3 | 14.7±1.2 | 14±1 | 12.2±1.8 | 0.003^¶^ |
| **N. of platelet counts (x10^9^/l)*** | 228±56 | 220±44 | 230±60 | 255±80 | NS |
| **N. of leucocytes counts (x10^9^/l)*** | 6785±1967 | 7073±1821 | 6523±1811 | 7122±3442 | NS |
| **Serum tryptase (ng/ml)*** | 86.2 ± 153.6 | 36.9±50.3 | 71.4±60.7 | 433±380 | <0.001^¶^ |
| **Altered TPSAB1 genotype** | 13 (13%) | 7 (18%) | 3 (5.5%) | 3 (33%) | 0.009^§^ |
| **LDH (U/l)** | 196.1±82.2 | 218±88 | 187±73 | 148.1±88 | 0.042^¶^ |
| **SAP (U/l)** | 101.2±173.9 | 71.±20 | 75.3±23 | 426.7±543.7 | <0.001^‡ §^ |
| **Serum β-2-microglobulin (µl/ml)** | 3.7±15.9 | 5.8±25 | 2.1±0.5 | 3.7±1.3 | <0.001^‡ §^ |
| **D816V *KIT* mutation** | 113 (98%) | 43 (96%) | 61 (100%) | 9 (100%) | NS |
| **Other gene mutations**  ***ASXL1***  ***CSMD1***  ***DNAH2***  ***DNMT3A***  ***SAR***  ***SETBP1***  ***SRSF2***  ***TET2*** | 2 (3%)  2 (3%)  1 (2%)  2 (3%)  1 (2%)  1 (2%)  1 (2%)  2 (3%) | 1 (6%)  0  0  0  0  0  0  0 | 0  2 (3%)  1 (2%)  2 (3%)  1 (2%)  1 (2%)  0  1 (2%) | 1 (14%)  0  0  0  0  0  1 (14%)  1 (14%) | NS |
| **% BMMC^*^ by FCM** | 0.35%± 0.9 | 0.11%±0.12 | 0.29%±0.41% | 1.99±2.6% | 0.015^⸋^ |
| **Follow up (months)^*^** | 118±86 | 93±44 | 127±74 | 195±210 | NS |
|  |  |  |  |  |  |

**Table 2 SuppInfo.** Distribution of healthy donors (HD) (n=32) and systemic mastocytosis (SM) patients per diagnostic subtype of the disease (n=115) according to the type of assays performed in this study.

| **Assay type** | **Healthy donors**  **n=32** | **SM**  **n=115** | **SM patients** | | |  |
| --- | --- | --- | --- | --- | --- | --- |
|  |  |  | **BMM**  **n=45** | **ISM**  **n=61** | **ASM**  **n=9** |  |
| **Immunophenotypic studies** | 12 | 70 | 24 | 39 | 7 |  |
| **Ex vivo cytokine production** | 16 | 53 | 20 | 28 | 5 |  |
| **Soluble cytokine plasma levels** | 6 | 52 | 19 | 28 | 5 |  |
| **Soluble CCL2 and EMR2 plasma levels** | 15 | 57 | 20 | 32 | 5 |  |
| **Soluble CXCR2, SAA1 and TLR2 plasma levels** | 10 | 30 | 9 | 17 | 4 |  |
| **TPSAB1 genotype** | 14 | 103 | 40 | 54 | 9 |  |

Results expressed as number of cases. SM, systemic mastocytosis; BMM, bone marrow mastocytosis; ISM, indolent systemic mastocytosis; ASM, aggressive systemic mastocytosis; CCL2, C-C motif chemokine ligand 2; EMR2, epidermal growth factor-like module–containing mucin-like hormone receptor–like 2; CXCR2, C-X-C motif chemokine receptor 2; SAA1, serum amyloid A1; TLR2, toll like receptor 2.

**Table 3 SuppInfo.** List of monoclonal antibody reagents used for immunophenotypic analysis of different populations of blood monocytes and dendritic cells from SM patients and healthy donors.

| Marker | Clone | Fluorochrome | Source |
| --- | --- | --- | --- |
| CD1 | F10/21A3 | BBB515 | BD Biosciences^a^ |
| CD3 | UCHT1 | BV711 | BD Biosciences |
| CD5 | UCHT2 | BV510 | BD Biosciences |
| CD14 | MφP9 | APCH7 | BD Biosciences |
| CD16 | 3G8 | BV786 | BD Biosciences |
| CD19 | SJ25C1 N | BV711 | BD Biosciences |
| CD33 | P67.6 | BV605 | BD Biosciences |
| CD33 | P67.6 | PECy7 | BD Biosciences |
| CD34 | 581 | PECF594 | BD Biosciences |
| CD36 | CLB-IVC7 | PerCPCy5.5 | Immunostep^b^ |
| CD45 | HI30 | OC515 | BD Biosciences |
| CD45 | HI30 | AF700 | BD Biosciences |
| CD56 | NCAM16.2 | BV711 | BD Biosciences |
| CD62L | DREG56 | BV650 | Biolegend^c^ |
| CD123 | 7G3 | BV650 | BD Biosciences |
| CD141 | 1A4 | BV421 | BD Biosciences |
| CD182 | 6C6 | APC | BD Biosciences |
| CD192 | K036C2 | BV605 | Biolegend |
| CD282 | 11G7 | BV510 | BD Biosciences |
| CD300e (IREM2) | UP-H2 | APC | Immunostep |
| CD303 | AC144 | APC | Miltenyi Biotec^d^ |
| CD312 | REA302 | PE | Miltenyi Biotec |
| HLADR | G46-6 | PECF594 | BD Biosciences |
| HLADR | G46-6 | BV711 | BD Biosciences |
| IL1β | AS10 | PE | BD Biosciences |
| IL6 | MQ2-6A3 | PE | BD Biosciences |
| IL8 | E8N | PECy7 | Biolegend |
| IL10 | JES3-9D7 | PECy7 | Biolegend |
| IL12 | C8.6 | BV421 | BD Biosciences |
| IL13 | JES10-5A2 | PerCPCy5.5 | Biolegend |
| TGFβ | TW4-9E7 | BV421 | BD Biosciences |
| TNFα | Mab11 | PerCPCy5.5 | Biolegend |
| FcERI | AER-37 | FITC | Thermo Fisher^e^ |
| FcERI | AER-37 | PE | Thermo Fisher |
| PAR-2 | 344222 | APC | R&D Systems^f^ |
| Slan | DD.1 | PE | Miltenyi Biotec |

Abbreviations: AF700, Alexa Fluor 700; APC, allophycocyanin; APCH7, allophycocyanin-Hilite®7 BB515, Brilliant Blue 515; BV421, Brilliant Violet 421; BV605, Brilliant Violet 605; BV650, Brilliant Violet 650; BV711, Brilliant Violet 711; FITC, fluorescein isothiocyanate; PacB, Pacific Blue; PE, phycoerythrin; PECF594, PerCPCy5.5, peridinin chlorophyll protein-cyanin 5.5; PECy7, phycoerythrin-cyanin 7; PacO, Pacific Orange; ^a^BD Biosciences (San Jose, CA); ^b^Immunostep (Salamanca, Spain); ^c^Biolegend (San Diego, CA); ^d^Miltenyi Biotec (Cologne, Germany); ^e^Thermo Fisher (Waltham, MA).; ^f^R&D Systems (Minneapolis, MN).

|  | Anaphylaxis | | Osteoporosis | | Pruritus  **Table 4 SuppInfo.** Relationship between the presence of symptoms and the distribution of distinct populations of monocytes and dendritic cells in blood of systemic mastocytosis (SM) patients (n=70). | | Gastrointestinal symptoms | | P value |
| --- | --- | --- | --- | --- | --- | --- | --- | --- | --- |
|  | Yes  n=37 | No  n=33 | Yes  n=11 | No  n=59 | Yes  n=36 | No  n=34 | Yes  n=21 | No  n=49 |  |
| N. of total pb monocytes (cells/μl) | 397  (175-3458) | 336  (103-2011) | 363  (205-749) | 377  (103-3458) | 348  (103-701) | 408  (150-3458) | 397  (103-2011) | 347  (150-3458) | * |
| N. of PB classical monocytes (cells/μl) | 359  (169-3349) | 298  (84-1995) | 318  (172-592) | 329  (84-3349) | 304  (84-659) | 367  (136-3349) | 348  (84-1995) | 313  (136-3349) | * |
| N. of PB intermediate monocytes (cells/μl) | 17  (2-85) | 12  (3-35) | 14  (6-85) | 13  (2-58) | 13  (36-2) | 15  (7-85) | 14  (5-33) | 13  (2-85) | * |
| N. of PB non-classical monocytes (cells/μl) | 29  (4-71) | 19  (4-46) | 22  (12-71) | 23  (4-67) | 21  (4-51) | 28  (4-71) | 22  (4-47) | 23  (4-71) | ***  † |
| N. of total dendritic cells (cells/μl) | 24  (7-55) | 14  (3-46) | 23  (11-36) | 17  (3-55) | 17  (3-55) | 23  (5-46) | 20  (3-55) | 18  (5-46) | *** |
| N. of PB myeloid DC (cells/μl) | 16  (4-41) | 9  (2-43) | 16  (8-24) | 11  (2-43) | 11  (2-41) | 14  (2-43) | 13  (2-43) | 11  (2-31) | ** |
| N. of PB plasmocytoid DC (cells/μl) | 8  (2-18) | 4  (1-14) | 9  (3-18) | 6  (1-17) | 5  (0.5-17) | 7  (2-18) | 5  (0.6-14) | 6  (1-18) | *** |
| N. of PB axl dc (cells/μl) | 0.5  (0-2) | 0.3  (0-0.6) | 0.3  (0-0.9) | 0.4  (0-2) | 0.4  (0-2) | 0.4  (0-2) | 0.3  (0-0.7) | 0.4  (0-2) | *** |

Results expressed as median values and range between brackets. Presence vs absence for * anaphylaxis, ‡ osteoporosis, † pruritus, § gastrointestinal symptoms. PB, peripheral blood; DC, dendritic cells. P-values: * <.05, ** <.010, *** < .001, † <.05, †† <.010, ††† < .001.

| **Table 5 SuppInfo.** Relationship between the presence of the *KIT* D816V mutation in blood monocytes of systemic mastocytosis (SM) patients (n=25) and the distribution of distinct populations of monocytes and dendritic cells and cytokine producing monocytes counts in blood. | ***KIT* D816V-mutational status of blood monocytes** | | |
| --- | --- | --- | --- |
|  | ***KIT* D816V^+^**  **(n=14)** | ***KIT* D816V^-^**  **(n=11)** | **P value** |
| **Diagnostic subtype of SM:** |  |  |  |
| BMM (n=10) | 3/14 (21%) | 7/11 (64%) | .032 |
| ISM (n=15) | 11/14 (79%) | 4/11 (36%) |  |
| **Number of monocytes and dendritic cells (DCs) in PB^*^:** |  |  |  |
| **N. of total PB monocytes (cells/ µl)** | 338  (254-363) | 369  (317-421) | NS |
| N. of PB classical monocytes (cells/ µl) | 291  (241-300) | 304  (260-348) | NS |
| N. of PB intermediate monocytes (cells/ µl) | 12  (7-35) | 26  (19-33) | NS |
| N. of PB non-classical monocytes (cells/ µl) | 25  (7-47) | 39  (38-39) | NS |
| **N of total dendritic cells** | 16  (11-24) | 17  (15-20) | NS |
| N. of PB myeloid DC (cells/ µl) | 9  (6.7 -16) | 13  (10-16) | NS |
| N. of PB plasmocytoid DC (cells/ µl) | 6.1  (4.5-8.7) | 4  (3.4-4.8) | NS |
| N. of PB AXL DC (cells/ µl) | 0.33  (0.07-0.37) | 0.30  (0.12-0.47) | NS |
| ***Ex vivo* cytokine production:** |  |  |  |
| N. of PB IL1β+ (cells/ µl) | 27  (0-222) | 34.47  (0-153) | NS |
| N. of PB IL6+ (cells/ µl) | 21  (2.9-76) | 13.01  (0-65.72) | NS |
| N. of PB IL8+ (cells/ µl) | 22  (3.9-87) | 27  (0.62-147) | NS |
| **Plasma levels:** |  |  |  |
| IL1β (pg/ml) | 4122  (91-9635) | 4163.  (204-7491) | NS |
| IL6 (pg/ml) | 15,698  (214-33,191) | 15301  (2722-31,395) | NS |
| IL8 (pg/ml) | 16,285  (1773-25,022) | 17,072  (5067-26,907) | NS |

Results expressed as number of cases/total cases (percentage) or as ^*^median values and range between brackets. BMM, bone marrow mastocytosis; ISM, indolent systemic mastocytosis; MC, mast cell; PB, peripheral blood; DC, dendritic cells.

|  | ***TPSAB1* genotype** | | |
| --- | --- | --- | --- |
|  | **HαT**  **(n=13)** | **Non-HαT**  **(n=90)** | **P value** |
| **Diagnostic subtype of SM:** |  |  |  |
| BMM (n=40) | 7/40 (18%) | 33/40 (83%) | .033 |
| ISM (n=54)  ASM (n=9) | 3/54 (6%)  3/9 (33%) | 51/54 (94%)  6/9 (67%) |  |
| **Number of monocytes and dendritic cells (DCs) in PB^*^:** |  |  |  |
| **N. of total PB monocytes (cells/ µl)** | 428  (313-701) | 348  (103-3458) | .036 |
| N. of PB classical monocytes (cells/ µl) | 363  (260-659) | 298  (8-3349) | .02 |
| N. of PB intermediate monocytes (cells/ µl) | 21  (9-36) | 13  (0.2-85) | NS |
| N. of PB non-classical monocytes (cells/ µl) | 33  (10-52) | 25  (7-47) | NS |
| **N of total dendritic cells** | 23  (10-38) | 14  (3-46) | NS |
| N. of PB myeloid DC (cells/ µl) | 16  (7-22) | 10  (2-31) | 0.045 |
| N. of PB plasmocytoid DC (cells/ µl) | 7  (2-17) | 5  (0.2-18) | NS |
| N. of PB AXL DC (cells/ µl) | 0.4  (0.05-0.95) | 0.4  (0.03-2.1) | NS |
| ***Ex vivo* cytokine production:** |  |  |  |
| N. of PB IL1β+ (cells/ µl) | 34.47  (0-153.43) | 13  (0-222) | NS |
| N. of PB IL6+ (cells/ µl) | 13.01  (0-65.72) | 11  (0-76) | NS |
| N. of PB IL8+ (cells/ µl) | 26.66  (0.62-147.29) | 17  (0.5-147) | NS |
| **Plasma levels:** |  |  |  |
| IL1β (pg/ml) | 4688  (168-7491) | 1413  (41-9932) | NS |
| IL6 (pg/ml) | 15301  (3042-28900) | 13473  (214-33191) | NS |
| IL8 (pg/ml) | 12015  (8806-26907) | 14997  (1773-28246) | NS |

**Table 6 SuppInfo.** Relationship between the presence of an altered *TPSAB1* genotype in systemic mastocytosis (SM) patients (n=103) and the distribution of distinct populations of blood monocytes, dendritic cells, cytokine producing monocytes and sytokine levels in plasma.

Results expressed as number of cases/total cases (percentage) or as ^*^median values and range between brackets. BMM, bone marrow mastocytosis; ISM, indolent systemic mastocytosis; MC, mast cell; PB, peripheral blood.

**Figure_1_SuppInfo**. Distribution of spontaneous (ex vivo) cytokine (IL1β, IL1γ and TGFβ) producing monocytes (A) and cytokine plasma levels (B), and the correlation among them (C), in healthy donors (HD) vs systemic mastocytosis (SM) patients classified according to the distinct diagnostic subtypes of the disease. Notched-boxes extend from 25^th^ to 75^th^ percentile values, the lines in the middle and vertical lines correspond to median values and the 10^th^ and 90^th^ percentiles, respectively, while dots represent each individual case analyzed. The percentage depicts the percentage of SM patients with numbers above 95% of the HD. P-values: * <.05, ** <.010, *** < .001.

**Figure_2_SuppInfo**. CCL2, CXCR2, SAA1 and EMR2 plasma levels (A-H) in healthy donors (HD) vs systemic mastocytosis (SM) patients (A, C, E and G) classified according to the distinct diagnostic subtypes of the disease (B, D, F and H). Notched-boxes extend from 25^th^ to 75^th^ percentile values, the lines in the middle and vertical lines correspond to median values and the 10^th^ and 90^th^ percentiles, respectively, while dots represent each individual case analyzed. * vs HD, ▲ vs BMM, # vs ISM. P-values: * <.05, ** <.010, *** < .001, ▲ <.05, ▲▲ <.010, ▲▲▲ < .001, # <.05, ## <.010, ### < .001. BMM, bone marrow mastocytosis; ISM, indolent systemic mastocytosis; ASM, aggressive systemic mastocytosis.

**Figure_3_SuppInfo**. Schematic graphical representation of the main results of this study associated with increased spontaneous cytokine production by blood monocytes, elevated cytokine plasma levels, and changes in the distribution of monocyte and dendritic cells observed in SM patients vs healthy donors, and their potential pathophysiologic implications.

**(A) Spontaneous *ex vivo* cytokine producing blood monocytes**

**(B) Cytokine levels in plasma**

**(C) Correlation between plasma levels and spontaneous (*ex-vivo)* cytokine producting blood monocyte counts**

Figure 1 SuppInfo_Pérez-Pons *et al.*

**(A) (B) (C) (D)**

**(E) (F) (G) (H)**

Figure 2 SuppInfo_Pérez-Pons *et al.*


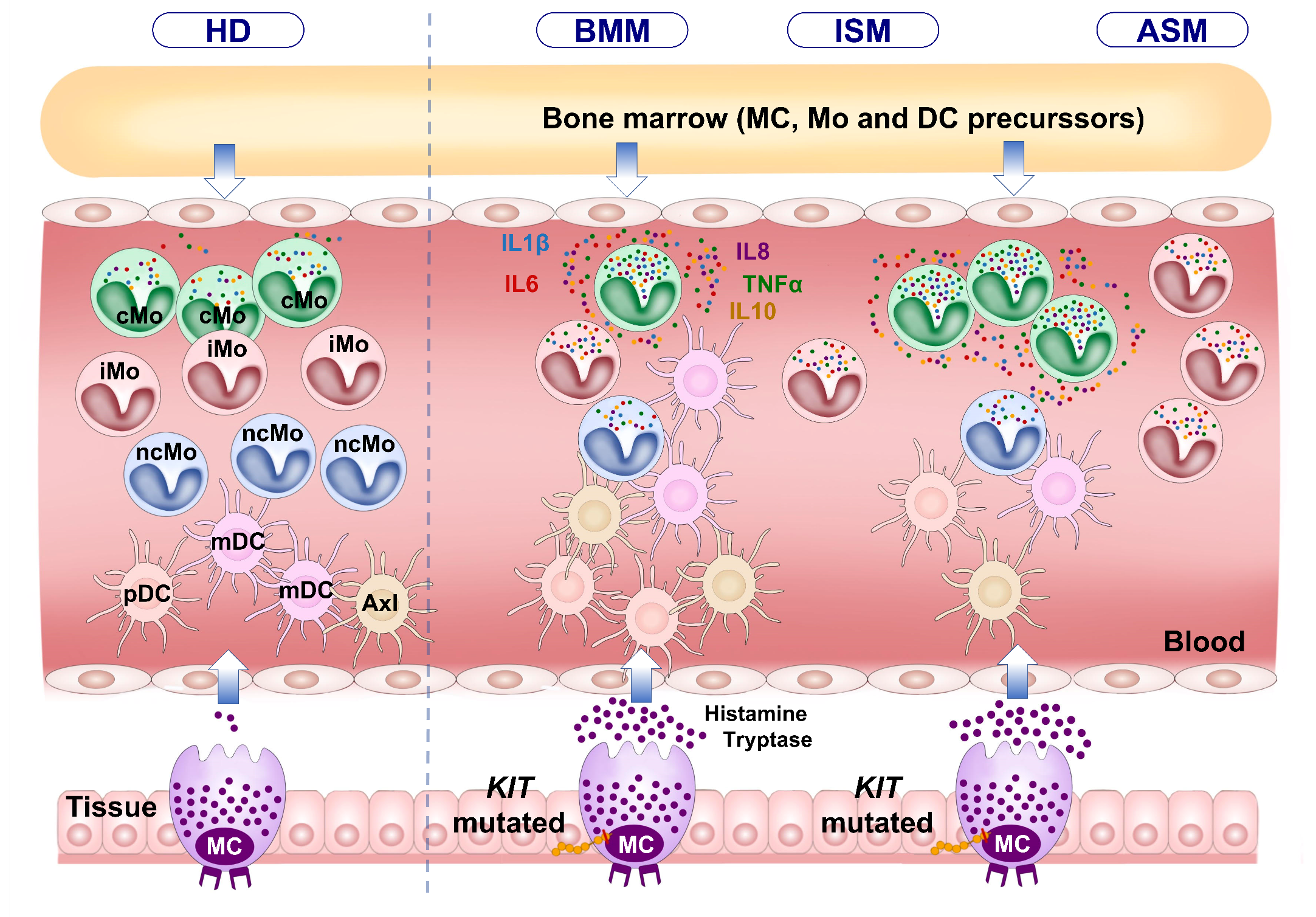


Figure 3 SuppInfo_Pérez-Pons *et al.*
